# Beyond conventional bounds: Surpassing system limits for stereotactic ablative (SAbR) lung radiotherapy using CBCT‐based adaptive planning system

**DOI:** 10.1002/acm2.14375

**Published:** 2024-05-07

**Authors:** Yesenia Gonzalez, Justin Visak, Luke Overman, Chien‐Yi Liao, Allen Yen, Tingliang Zhuang, Bin Cai, Andrew Godley, Yuanyuan Zhang, Robert Timmerman, Puneeth Iyengar, Kenneth Westover, David Parsons, Mu‐Han Lin

**Affiliations:** ^1^ Department of Radiation Oncology University of Texas Southwestern Medical Center Dallas Texas USA; ^2^ Department of Radiation Oncology Memorial Sloan Kettering Cancer Center New York City New York USA; ^3^ Medical Artificial Intelligence and Automation Laboratory Department of Radiation Oncology University of Texas Southwestern Medical Center Dallas Texas USA

**Keywords:** adaptive radiotherapy, lung SAbR, treatment‐planning

## Abstract

**Purpose:**

Online adaptive radiotherapy relies on a high degree of automation to enable rapid planning procedures. The Varian Ethos intelligent optimization engine (IOE) was originally designed for conventional treatments so it is crucial to provide clear guidance for lung SAbR plans. This study investigates using the Ethos IOE together with adaptive‐specific optimization tuning structures we designed and templated within Ethos to mitigate inter‐planner variability in meeting RTOG metrics for both online‐adaptive and offline SAbR plans.

**Methods:**

We developed a planning strategy to automate the generation of tuning structures and optimization. This was validated by retrospective analysis of 35 lung SAbR cases (total 105 fractions) treated on Ethos. The effectiveness of our planning strategy was evaluated by comparing plan quality with‐and‐without auto‐generated tuning structures. Internal target volume (ITV) contour was compared between that drawn from CT simulation and from cone‐beam CT (CBCT) at time of treatment to verify CBCT image quality and treatment effectiveness. Planning strategy robustness for lung SAbR was quantified by frequency of plans meeting reference plan RTOG constraints.

**Results:**

Our planning strategy creates a gradient within the ITV with maximum dose in the core and improves intermediate dose conformality on average by 2%. ITV size showed no significant difference between those contoured from CT simulation and first fraction, and also trended towards decreasing over course of treatment. Compared to non‐adaptive plans, adaptive plans better meet reference plan goals (37% vs. 100% PTV coverage compliance, for scheduled and adapted plans) while improving plan quality (improved GI (gradient index) by 3.8%, CI (conformity index) by 1.7%).

**Conclusion:**

We developed a robust and readily shareable planning strategy for the treatment of adaptive lung SAbR on the Ethos system. We validated that automatic online plan re‐optimization along with the formulated adaptive tuning structures can ensure consistent plan quality. With the proposed planning strategy, highly ablative treatments are feasible on Ethos.

## 1 INTRODUCTION

Radiotherapy plays a significant role in the treatment of medically inoperable lung cancer patients.^1^ For early‐stage non‐small‐cell patients, stereotactic ablative radiation therapy (SAbR) is now the standard‐of‐care, offering durable local control rates with one to five treatments.^2^, ^3^, ^4^ Historically, the gold standard for these patients was forward planning using non‐coplanar 3D conformal RT (3DCRT). However, with the widespread application of SAbR, more complex geometries benefit from inverse treatment planning. Several studies have shown that techniques like intensity‐modulated RT (IMRT) or volumetric modulated arc therapy (VMAT) can achieve similar plan quality with minimum sacrifice of dose conformality.^5^, ^6^ While 3DCRT is simpler, IMRT and VMAT allow for dose escalation without excess toxicity to organs‐at‐risk (OARs). Despite advancements of delivery techniques, patients were historically treated with the same reference plan throughout, using image‐guidance but not accounting for inter‐fractional anatomy or setup changes.

Recently, online adaptive radiotherapy systems have emerged, allowing more customization and dosimetric improvement by modifying each fraction's plan based on daily anatomy.^7^ This signifies the next major advance in non‐small cell lung cancer SAbR treatment. One commercially available cone‐beam CT‐based online adaptive treatment platform is the Varian Ethos system (Varian Medical Systems, Palo Alto, CA, USA). Ethos shares identical hardware with the clinically validated Varian Halcyon linac, which has demonstrated safe lung SAbR delivery.^8^ Ethos differs in having an integrated offline/online treatment‐planning system, functionally distinct from other commercial treatment planning system (TPS). The Ethos planning system streamlines both pre‐plan and online adaptive plan generation using the Intelligent‐Optimizer‐Engine (IOE).^9^, ^10^ The IOE removes direct planner optimization control, introducing a new ‘Dose Preview’ tab for planners to input and rank clinical priorities (i.e., P1–P4). The placement and ranking of clinical priorities is used in lieu of manually weighting the optimization criteria, typical of the optimizer in a conventional TPS. The IOE communicates these goals to the optimizer and blinds the planner in the final optimization goals and priority during final reference plan generation. This leads to the Ethos TPS being more streamlined, but simultaneously more rigid in its design than conventional TPSs where planners are offered more flexibility and tools to create a given treatment plan. The IOE has been validated to deliver high quality plans with careful selection of clinical goals. At the time of preparation of this manuscript minimal guidance exists for lung SAbR reference plan generation.^11^, ^12^, ^13^, ^14^ The Ethos IOE was originally designed for conventional treatments and contains built‐in plan quality constraints, among them, one of which is a hot spot controlling structure. Therefore, it is crucial to provide clear guidance for the lung SAbR treatments due to the qualities differentiating SAbR and conventional plans. For SAbR plans, the planning target volume (PTV) is made using a margin off the internal target volume (ITV) and the aim is to deliver a highly conformal dose per fraction with sharp intermediate dose gradients.^15^ Additionally, the planner must also drive the maximum dose to be centered in the tumor volume for a plan to be considered high‐quality. It is well‐documented that plan quality is highly correlated to inter‐planner variability and a novice planner may not be able to meet SAbR criteria.^16^, ^17^ Hence, due to the rigidity of the Ethos TPS and the inherent intent for conventional treatments, the use of our lung SAbR planning template has allowed us to consistently create robust, clinically acceptable SAbR plans.

Delivering lung SAbR on Varian Ethos is technically rigorous and requires specialized training and staff to consistently generate robust treatments. Moreover, with the blinding of optimization parameters in the final generation, it is critical that a simple and well‐understood planning strategy is developed. A robust treatment is highly dependent on initial reference plan quality and is crucial as an adaptive program minimizes inter‐planner variability. The IOE limits the conventional optimization techniques due to online computational requirements and streamline ability. (e.g., large optimization structures, customized normal tissue objective fall‐off). Therefore, it is incumbent to develop a planning strategy with adaptive‐mindful optimization goals thereby maximizing plan quality. To offer a safe treatment, the plan must conform to standards set forth by the Radiation Therapy Oncology Group (RTOG) Reports for centrally and peripherally located lung lesions.^18^, ^19^, ^20^ The purpose of this study is to evaluate the feasibility of using IOE to automatically generate lung SAbR and develop a robust planning workflow for online adaptive lung SAbR treatments that will allow both a novice and highly skilled planner to generate similar plans. Moreover, we validate the use of novel adaptive‐specific optimization tuning structures versus conventional planning wisdom and compare the dosimetric results to population based RTOG metrics for both online and offline plans.

## 2 MATERIALS AND METHODS

### 2.1 Patient cohort

Institutional Review Board approval was obtained to complete this study. Our Ethos based adaptive program started service in June 2021 for both conventional and SAbR lung patients. This study retroactively selected 35 lung SAbR patients treated on Ethos. All patients were treated with a prescription ranging from 50 to 60 Gy in either 3 or 5 daily adaptive fractions. The patient demographics are summarized in Table 1. Patients ranged from 17 to 85 years of age, with the mean age being 67 years at time of treatment. All patients underwent a 4D CT simulation where a mini‐scan was first acquired to assess target motion. Any patients with target motion greater than 1–1.5 cm were applied compression.

**TABLE 1:** Demographics of patient cohort including all 35 retrospectively analyzed patients.

Demographics (%)					
Gender		Age		Ethnicity	
**Female**	58.7	**<50**	2.2	**White**	56.5
		**50–60**	8.7	**AA**	28.3
**Male**	41.3	**60–70**	54.3	**Hispanic**	13
		**70+**	34.8	**Asian**	2.2

Diagnosis (%)			
Stage		Location	
**I**	54.3	**LUL**	41.3
**II**	6.5	**RUL**	28.3
**III**	8.7	**LLL**	8.7
**IV**	3.6	**RLL**	17.4
		**Other**	4.3

Abbreviations: LLL, left lower lobe; LUL, left upper lobe; RLL, right lower lobe; RUL, right upper lobe.

### 2.2 Lung SAbR planning strategy

The Ethos simulation workflow is identical to conventionally treated patients on a C‐arm linac. Patients are immobilized within a stereotactic body frame (Elekta AB, Stockholm, Sweden) and undergo a 10‐phase 4DCT and free‐breathing CT for simulation. The maximum intensity projection (MIP) image is used to create the ITV, then typically expanded 5 mm to generate PTV. The ITV on MIP is propagated to average CT and used for treatment planning.

The Ethos TPS (Ethos V1.1) leverages the IOE to automate the plan optimization process. However, in our initial review of the process it was observed the automated 12‐IMRT fields generates a homogenous target dose causing unacceptable intermediate dose spill that violates RTOG gradient index (GI) criteria.

**FIGURE 1:**
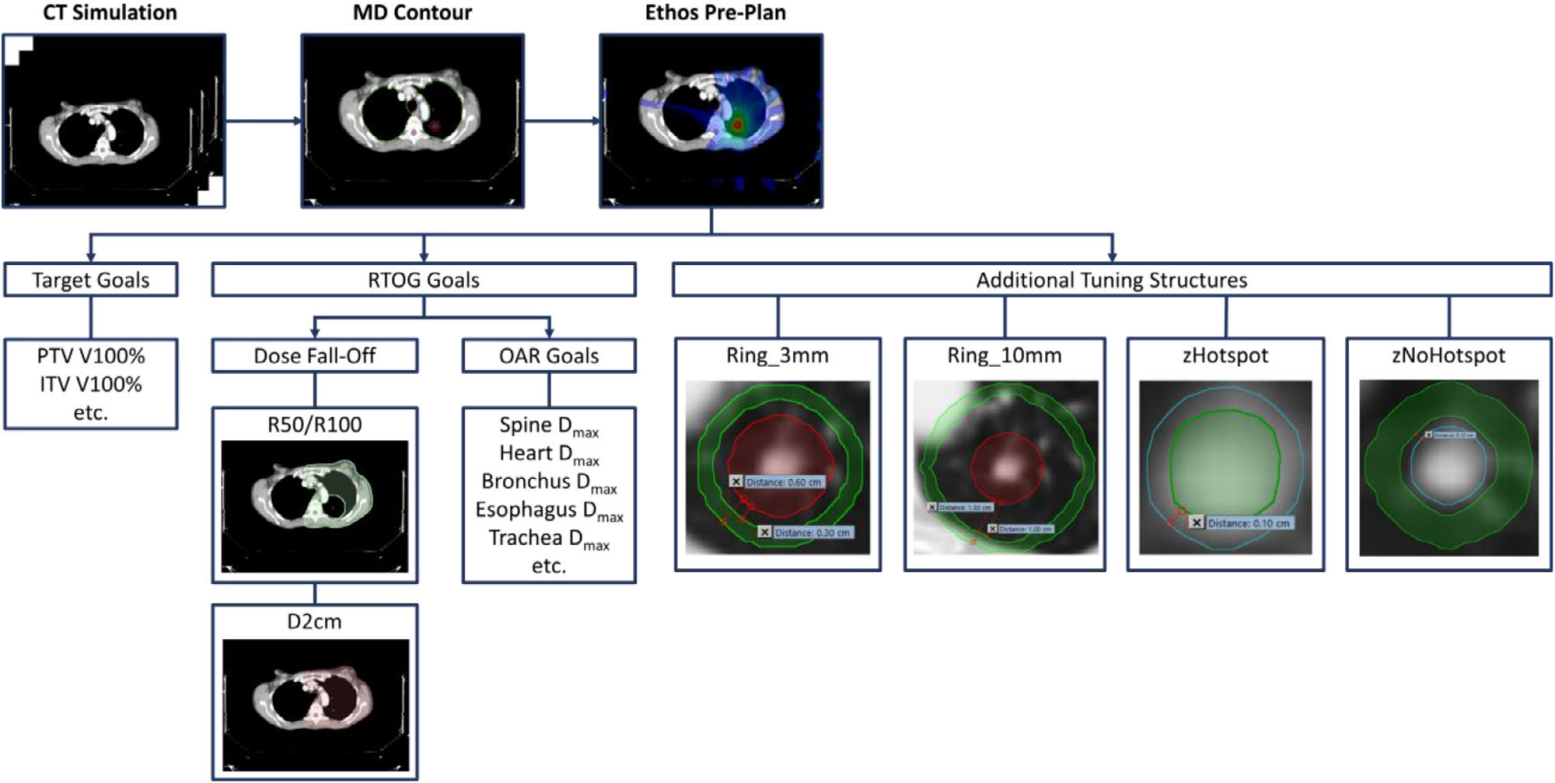
Workflow of adaptive treatment planning process. patient is sent for CT simulation, the physician contours the OARs and targets, planners generate a reference plan within the Ethos treatment planning system to ensure subsequent adaptive plans meet both target and RTOG goals. Additional tuning structures are shown in green, PTV in red, and ITV in blue in sub‐figures. Margin formulas are defined in Table 2. ITV, internal target volume; OARs, organs‐at‐risk; PTV, planning target volume; RTOG, radiation therapy oncology group.

This prompted the development of a simple and robust planning strategy that can generate SAbR‐like dose distribution consistently (Figure 1). The Ethos system is a template‐based TPS, site, laterality and dose specific templates for lung SAbR treatments were generated (e.g., centrally‐located 50 Gy in five, peripherally located 54 Gy in three). In general, the templates follow RTOG 0813/0618 dosimetric criteria and OAR dose is further reduced using an in‐house developed dose prediction model. In addition to the dose volume histogram (DVH) metrics, we also deploy novel adaptive tuning structures that are used to improve plan quality: (1) centralized target sub‐volume maximum dose guidance structure, (2) inner ring to ensure high dose conformity (Ring_3 mm), (3) outer ring to push intermediate fall‐off (Ring_10 mm), (4) the patient's body structure subtracting a 2 cm expansion of the PTV (D2cm). These structures are derived based on daily anatomical contours and automatically update with anatomy changes (see Table 2). The addition of these tuning structures is necessary due to the limited features of Ethos compared to a traditional TPS, such as Eclipse (Varian Medical Systems Inc., Palo Alto, CA, USA). More specifically, the Ethos TPS has an automatic normal tissue objective (NTO) that is unable to be adjusted and the addition of the tuning structures is a work‐around to achieve our clinical goals. Specifically for our cohort of lung SAbR patients, all treatments plans were made using a beam arrangement of 9 to 13 fields (average of 10 fields), with a minimum of one contralateral beam that goes through anatomy visible on the cone‐beam CT (CBCT). All plans included in this study contained a single target and were normalized to 95% PTV coverage.

**TABLE 2:** Definition of Structures used in reference planning template and general priority level given to each structure.

Structures	Formula	Goal (3fx)	Goal (5fx)	Order
**Hard constraint OAR**	–	MD defined clinical goal		Top of P1
**zHotSpot**	ITV – 2 mm*	V110% > 90%		P1
**ITV coverage**	–	V100% > = 99%		P1
**PTV coverage**	–	V100% > = 95%		P1
**PTV V95**	–	V95% > = 99%		P1
**Tier1 OAR**	–	MD defined clinical goal and/or RTOG dose limit		P1
**zNoHotSpot**	PTV‐(ITV+1 mm)	D0.03cm^3^ < 110%		P1
**Ring_**3 mm	PTV: −0.3 cm inner, 0.6 cm outer	D0.03cm^3^ < 93% to 98%	D0.03cm^3^ < 83% to 93%	P1
**Ring_**10 mm	PTV: −1.0 cm inner, 1.3 cm outer	D0.03cm^3^ < 65% to 83%	D0.03cm^3^ < 50% to 75%	P1
**Humanbody**	Patient body without frame	V100%RX dose < = RTOG R100 volume limit V50%RX dose < = RTOG R50 volume limit		P1
**D2cm**	HumanBody‐(PTV+2 cm)	D0.03cm^3^ < = RTOG dose limit		End of P1
**Tier2 OARs**	–	RTOG dose limit		P2

Abbreviations: ITV, internal target volume; MD, medical doctor; OAR, organ‐at‐risk; PTV, planning target volume; RTOG, radiation therapy oncology group.

Objectives are ordered based on a priority level ranging from 1 to 4, or “report value only” which has no effect on planning. Hard constraints will be listed within priority, with the remaining objectives placed in priority 2−4. An initial dose preview assuming nine equidistant fields is dynamically updated and shown to the planner to help guide ordering of objectives within the priority levels. Ultimately, the planner will adjust the objectives to simultaneously meet the MDs constraints and achieve the highest plan quality possible. Ethos workflow is explained in more detail in other literature.^11^ We evaluated the impact of our lung SAbR planning strategy on Ethos via three main components: (1) effectiveness of planning strategy in producing desirable dosimetry, (2) ITV volume change over treatment, and (3) robustness of adaptive plan quality.

#### 2.2.1 Evaluation of SAbR planning using automated tuning structures

Our approach to mitigating the inflexibility in the NTO within the Ethos TPS was to introduce tuning structures such as customized rings to control conformality and dose fall off. To evaluate the effectiveness of our approach, we compared RTOG dosimetric criteria for 20 patients planned with or without the adaptive tuning structures. Criteria included maximum dose any 2 cm away from PTV surface (D2cm), 100% isodose volume divided by PTV (conformity index [CI]), 50% isodose volume divided by PTV GI, maximum dose divided by prescription dose (heterogeneity index [HI]), target coverage and OAR maximum and volumetric constraints.

In addition to dosimetric data, the modulation factor and gamma‐passing rates of measurement‐based quality assurance were recorded for both sets of plans. Measurements were performed with a cylindrical acrylic phantom with a three‐dimensional array of 1386 diode detectors with 10 mm spacing (ARCCheck; Sun Nuclear Inc., Melbourne, FL, USA). Gamma‐passing rates were recorded via measurement based quality assurance and evaluated at 3%/2 mm, 2%/2 mm, and 1%/1 mm. Significance for all metrics were quantified using a one‐sided *t*‐test.

#### 2.2.2 Evaluation of ITV volumetric changes

We recorded the volume of the ITV for all 35 patients at initial CT simulation and compared to the first adaptive fraction to verify that the CBCT on the Ethos produced high enough quality images to accurately contour the ITV. Patients are typically treated within a week after CT simulation so it is anticipated that there will be insignificant volume changes. Ethos does not offer 4D capability; however, the on‐board CBCT scan time is 16 s (fast scan) – 30 s (standard scan), which exceeds the average breathing cycle. In addition to this comparison, we prospectively tracked the change in volume of the ITV over the course of treatment (three or five fractions) and compared it relative to the volume of the ITV drawn in the first adaptive fraction. Shrinkage of the ITV would be an indicator of effective treatment.

#### 2.2.3 Robustness of SAbR adapted plan quality

It is important to generate a reference plan of high‐plan quality; however, that does not guarantee a planner's goals will translate well to the online plan. As stated above, the Ethos system locks a planner's goals for online re‐optimization and does not allow any modification. To address the potential that our robust strategy may cause challenges online, we retrospectively analyze the adaptive plan quality that was delivered to the patients and compare to the IGRT scheduled plan on the daily anatomy that the system automatically generates. This was accomplished by evaluating whether or not the target and OAR clinical goals were met or below the reference planning criteria. In our clinical practice, we do not plan patient treatments without using our described methodology, therefore the frequency of the plans not utilizing the described technique is unavailable.

## 3 RESULTS

### 3.1 Effectiveness of SAbR planning strategy

**FIGURE 2:**
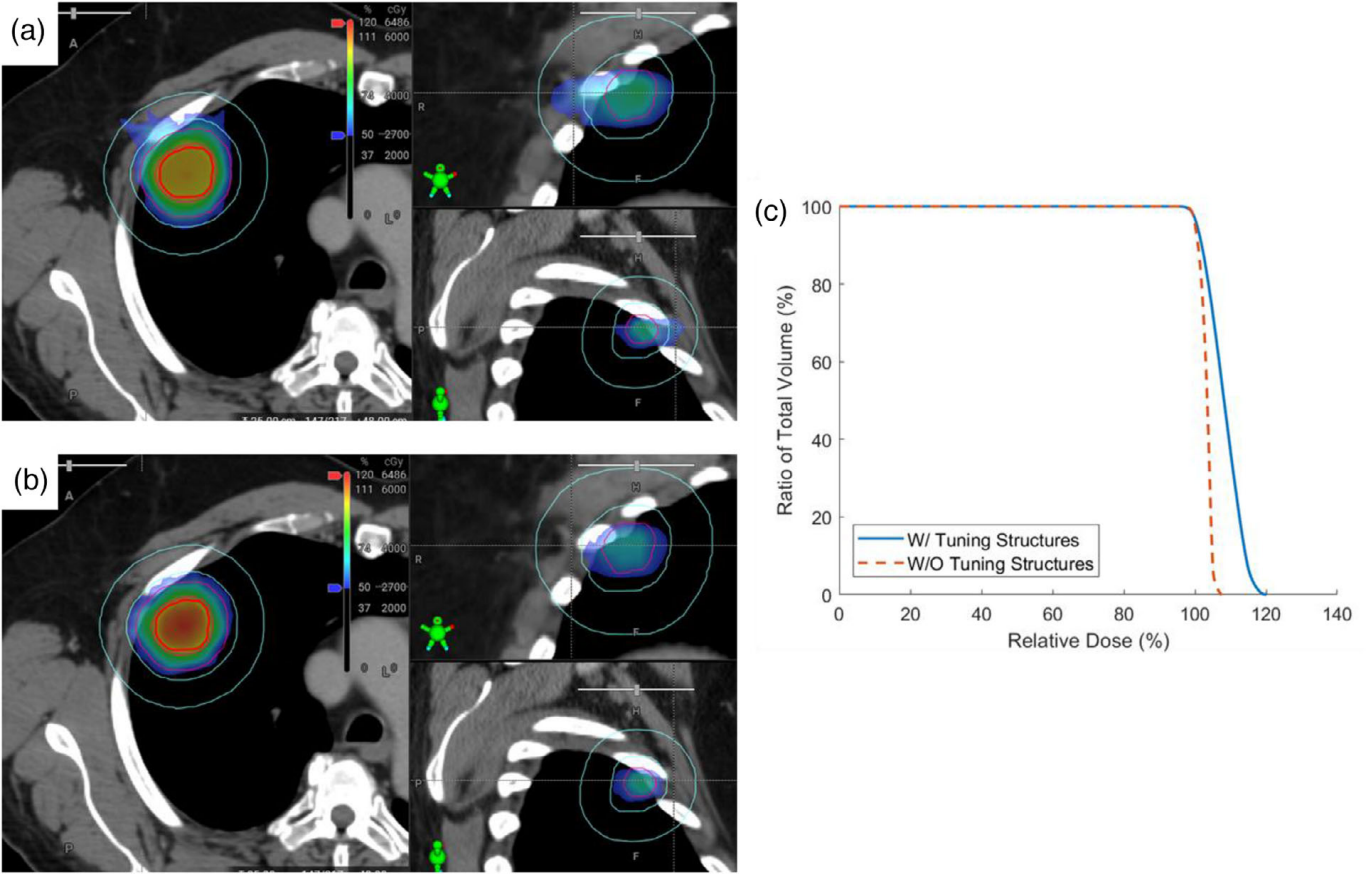
(a) Plan without the adaptive tuning structures; (b) plan with the adaptive tuning structures included; (c) DVH comparison of the two plan's PTV coverage, solid line corresponding to the plan with the adaptive tuning structures and dashed line corresponding to the plan without the adaptive tuning structures. No difference was seen outside the tail end of the DVH between the two plans. PTV, planning target volume.

To isolate the effects of the hotspot control and tuning rings to overcome the auto‐NTO, 20 patient plans were planned with both methods: with and without adaptive‐tuning structures. Visual comparison of the two methods is shown in Figure 2. We found that the addition of these structures increased the ITV V110% from 37.7% to 65% (*p* = 0.0018), and D50 increased by 2.74% (*p* < 0.001) when tuning structures were not used. They resulted in a tendency to improve the dose conformity (Figure 2a), on average, an improved GI by 1.87% (average GI of 4.65 and 4.74) and CI by 1.9% (average CI of 1.08 and 1.1), neither of which was statistically significant compared to the plan without the adaptive tuning structures. When evaluating HI between plans, the tuning structures increased HI on average from 1.17 to 1.20 (*p* = 0.02). All OAR D0.035cc were evaluated and constraints were met with no significant difference on both sets of plans.

Regarding plan deliverability, the tuning structures increased the average number of monitor units (MUs) per plan to 6383 ± 2603 compared to 5587 ± 2360 without tuning structures (*p* = 0.01). The average increase in MUs resulted in a higher modulation per plan and we calculated the average modulation factor (MU divided by dose in cGy) to be 5.2 ± 1.7 and 4.6 ± 1.8, with and without tuning structures, respectively (*p* = 0.01). The dose rate of the Ethos machines is 800 MU/minute, as such the higher MUs per plan resulted in a longer estimated beam‐on time of 7.98 min compared to 6.98 min (*p* = 0.01). Measurement based gamma analysis with 3%/2 mm criteria for all plans passed our institutional criteria of 90%. Average pass rate was 96.9 ± 2.2% and 95.3 ± 2.3% for plans with and without adapting tuning structures, respectively.

### 3.2 ITV change over treatment

We found no significant difference in ITV size between CT simulation and CBCT at the first adaptive fraction. In addition to this, we also tracked the ITV change over the course of treatment relative to the first adaptive fraction. We observed a decreasing trend in ITV over the course of adaptive treatment. When further analyzing this result, it was noticed that for seven patients with the largest ITV reduction throughout treatment demonstrated an average ITV reduction of 36% from the reference plan.

### 3.3 Robustness of SAbR adapted plan quality

**FIGURE 3:**
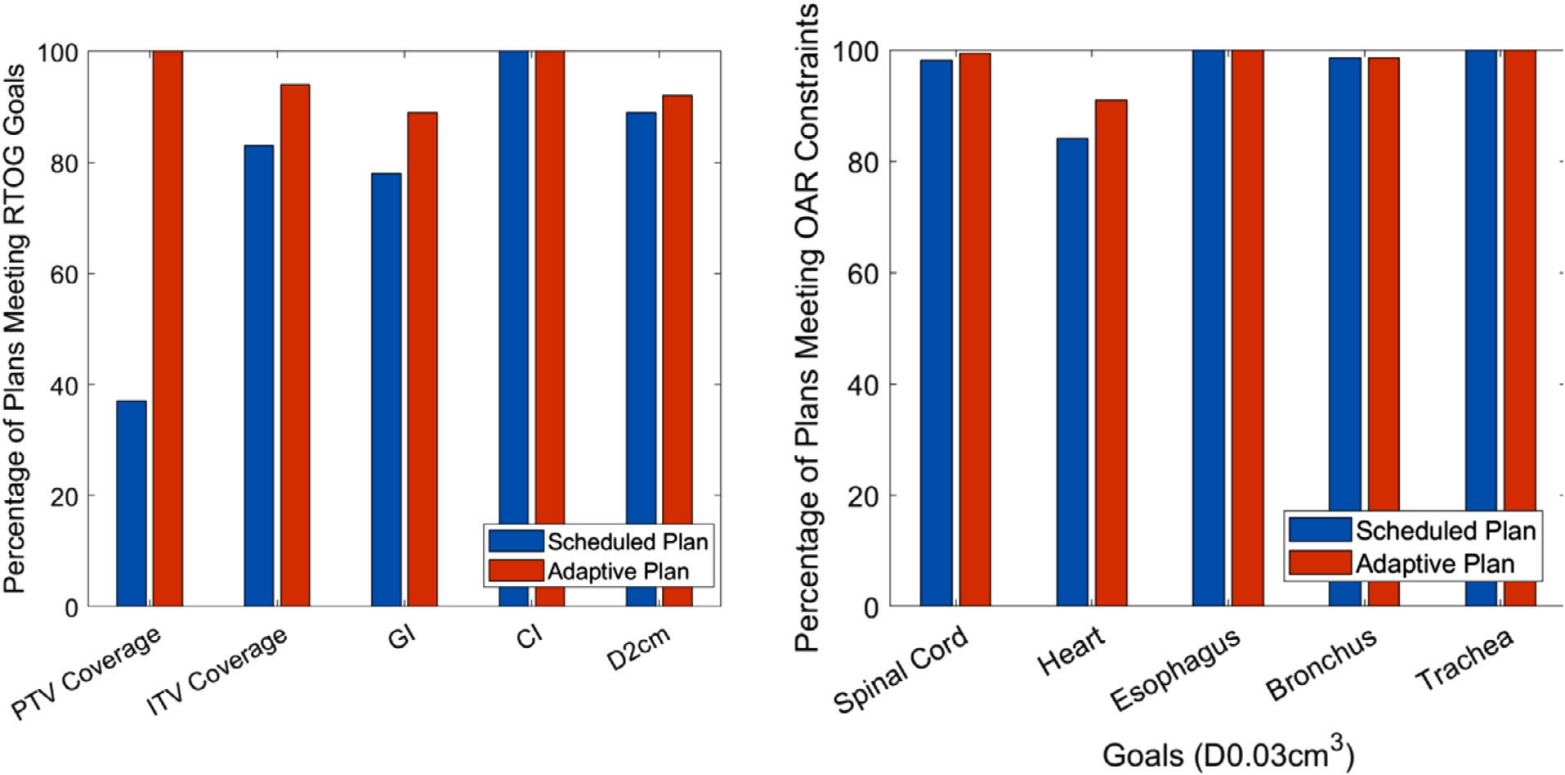
Percentage of scheduled and adaptive plans meeting both RTOG goals (left) and OAR constraints (right). OAR, organ‐at‐risk; RTOG, radiation therapy oncology group.

Robustness of our planning strategy was evaluated by comparing the frequency of meeting our initial planning objectives during the automatic online re‐plans for a total of 105 adaptive treatments (Figure 3). At the time of treatment, 92% of adaptive plans were selected by the physicians for treatment over the scheduled plans, which is the reference plan calculated on the anatomy of the day. All adapted plans met PTV coverage goals while only 37% of scheduled plans were able to meet PTV coverage. For ITV coverage, the adaptive plans met 99% ITV coverage 94% of the time while scheduled plan were able to meet ITV coverage only 83% of the time. Both plans met CI criteria 100% of the time while GI was met only 89% and 78% of the time for adaptive and scheduled plans, respectively. We found that the adapted plans improved GI and CI by 3.8% (*p* < 0.001) and 1.7% (*p* = 0.02), respectively, compared to the scheduled plan. The adaptive plan struggled to meet GI in adaptive sessions where GI was marginally met in the reference plan, especially in cases where the tumor volume changed in an unfavorable direction. The OAR constraints were based off RTOG guidelines; however, there were instances where they were modified per physician request. When comparing frequency of meeting OAR constraints, no difference was found between scheduled and adapted plans for the esophagus, bronchus, and trachea. The adapted plan met spinal cord constraints 100% of the time while the scheduled plan met 98% of the time. Similarly, heart constraints were met 90% of the time for adapted plans and 82% of the time for scheduled plans. All reference plans met heart constraints; however, instances of failure include cases where the PTV overlapped or shared a boundary with the heart.

## 4 DISCUSSION

x‐Ray guided online ART system offers automatic planning for the reference plan and utilizes the planning strategy employed in the reference plan phase for all subsequent online fractions. The auto‐planning feature has been previously demonstrated to result in high quality plans for the conventional fractionated treatments.^21^ We have developed and validated a safe and reproducible template for lung SAbR patients treated on the Varian Ethos machine. We demonstrated the value of the template and the adaptive tuning structures through the comparison of the plan with and without these tuning structures. The template allowed us to create clinically acceptable plans of a consistent quality that follow RTOG guidelines. This planning strategy is consistent with other treatment planning systems such as TomoTherapy (Accuray, USA) where the use of multiple tuning rings is deployed to control intermediate dose spillage.^22^ However, our proposed adaptive tuning structures are formulated based on ITV and can be dynamically adapted to account for gross tumor volume/ITV changes throughout the course of treatment based on careful margin selections.

When further comparing the dosimetry of the scheduled and adaptive plans, on average the adaptive plans outperformed the scheduled plans in terms of target coverage, sparing of the heart and spinal cord, and target indices (e.g., HI, D2cm, GI, CI). It is important to note that the quality of the plans during the adaptive sessions are dependent on and only reproducible up to the reference plan quality. One important drawback of the Ethos adaptive planning system is the reference plan constraints are fixed during adaption and will not currently allow for any modification throughout the course of treatment. A key example of this limitation is throughout treatment a PTV may shrink hence reducing the GI tolerance per RTOG criteria. Therefore, while the planner may originally input a constraint to meet the original GI criteria, the system will no longer push as aggressively as needed to control intermediate dose spillage with the new criteria.

We have also demonstrated the necessity of adaption with observation of significant change in tumor volume over the course of treatment for 50% of the cases evaluated in this study. In addition to anatomical changes, dosimetrically, the adaptive plan tended was preferentially chosen by the physician over the scheduled plan. More specifically, the adaptive plan was able to meet PTV coverage while also improving on qualities characteristic of a SAbR plan. There are selected instances due to minimal fraction‐to‐fraction change where the physician may select the scheduled plan in lieu of the adaptive plan. While the adaptive plan is potentially dosimetrically superior, in these cases it likely may be clinically insignificant. An important ongoing study at our institution is evaluating retrospective cases to help generate a prediction model that will guide the physician in selecting which fraction a lung SAbR patient would most benefit from adaption.

While many patients may preferentially experience changes in a reduced fraction regime (54 Gy in three treatments, our approach and the necessity of adaptation for lung SAbR is especially vital when patients are treated using a personalized ultrafractionated stereotactic adaptive radiotherapy (PULSAR). (Note that all the reported cases in this manuscript are non‐PULSAR cases.)^23^ PULSAR aims to shift the paradigm of radiotherapy treatments by combining effects of immunotherapy and radiological induced tumor microenviroment, proximal tissue, and systemic changes immediately following a ‘pulse’ of radiotherapy treatment.^23^ Rather than an every‐other‐day regime, PULSAR delivers ablative pulses separated on the order of weeks or months. Without an adaptive treatment unit, this would require patient to undergo an additional simulation prior to subsequent fractions to evaluate the need for a re‐plan. PULSAR patients treated on the Ethos machines with our robust planning approach would have a streamlined workflow that does not require additional simulations. At the time of treatment, the physician would modify the target based on the CBCT and since our adaptive tuning structures automatically adjust based on the target contours, there is no need to do a complete offline re‐plan.

## 5 CONCLUSION

Our institution has developed a robust and readily shareable planning strategy for the treatment of adaptive lung SAbR on the Ethos treatment unit. The original intent of the Ethos system was for the treatment of conventionally fractionated treatments, however, with simple and understandable adaptive tuning structures, highly ablative treatments are feasible. This study retrospectively evaluated a limited sample size of patients, but since the time of the original treatments, our institution has continued to see benefit of lung SAbR adaption. The template described in this work is sharable. Future work to predict adaption patterns and dosimetric improvement of dynamic planning constraints is ongoing.

## AUTHOR CONTRIBUTIONS

Yesenia Gonzalez and Mu‐Han Lin designed the study. Yesenia Gonzalez, Justin Visak, Chien‐Yi Liao, Luke Overman, Allen Yen performed data collection and analysis. Justin Visak and Yesenia Gonzalez drafted first iteration of manuscript. Mu‐Han Lin, Puneeth Iyengar, Kenneth Westover, David Parsons, Robert Timmerman, Yuanyuan Zhang, Andrew Godley, Bin Cai, Tingliang Zhuang provided clinical supervision and input to the project. All authors revised and approved the final manuscript.

## CONFLICT OF INTEREST STATEMENT

The authors declare no conflicts of interest.

## Data Availability

The data that support the findings of this study are available from the corresponding author upon reasonable request.
